# Effect of thermal treatment on microcracking characteristics of granite under tensile condition based on bonded-particle model and moment tensor

**DOI:** 10.1038/s41598-024-59470-0

**Published:** 2024-04-16

**Authors:** Jinsheng Zhao, Wei Sun, Hao Luo, Shunchuan Wu, Zhiqiang Hou

**Affiliations:** 1 State Grid Energy Hami Coal Power Co., Ltd Dananhu Second Mine, Hami, 839099 China; 2 Henan Key Laboratory for Green and Efficient Mining & Comprehensive Utilization of Mineral Resources, Henan Polytechnic University, Jiaozuo, 454002 China; 3 School of Energy Science and Engineering, Henan Polytechnic University, Jiaozuo, 454002 China; 4 Faculty of Land Resource Engineering, Kunming University of Science and Technology, Kunming, 650093 China

**Keywords:** Brazilian test, Thermal treatment, Yanshan granite, Microcracking, Moment tensor, Civil engineering, Computational science

## Abstract

It is known that the heterogeneity caused by thermally induced micro-cracks and thermal stress can affect the mechanical behavior of granite. The laboratory-scale tests have the intrinsic limitation of non-repeatability and lack of effective methods to characterize the interaction effect between thermal micro-cracks and thermal stresses. In this study, we demonstrate how advancements in particle bonded model and moment tensor can help better understand the roles of high temperature in weakening granite and thermally induced cracking process in Brazilian test. Our results show that the types of micro-cracks (intergranular, intragranular, and transcrystalline ones) are related to their thermal expansion coefficients of mineralogical compositions. The intergranular tensile micro-cracks are predominant during the heating and heating–cooling processes. An obvious weakening of granite and non-central initiation is associated with the heterogeneity caused by the thermal damage and thermal stress. We also quantitatively evaluate the thermal damage based on orientation distribution, *b*-value, and nature of the sources, which gives a new microcracking perspective on tensile characteristics subjected to high temperature.

## Introduction

Nuclear power is essential to address climate change while ensuring a stable energy supply to promote economic growth and protect the environment. The deep geological repository is suggested as one of the most reliable long-term storage methods^1–3^. URL (Underground Rock Laboratory, Canada)^4,5^, HRL (Hard Rock Laboratory, Sweden)^6,7^, ONKALO POSE experiment (Finland)^8,9^ and Beishan high-level radioactive waste repository (China)^10^ choice granite as the host natural rock due to its high strength, high thermal conductivity and low permeability. The damage and thermal stress caused by radiogenic heat affect the permeability characteristics of granite and can provide a rapid flow path for the migration of high radionuclides. The mechanical response of granite under thermo-mechanical coupling conditions becomes critical for similar deep underground projects.

The influence of temperature on the elastic modulus, Poisson’s ratio, and compressive strength of various granites have been widely studied, and were reviewed in detail by Dwivedi et al.^11^, Zhao^12^, and Sun et al.^13^. These experiments showed that granites are prone to generate the intergranular or intragranular micro-cracks at the mineral boundary or inside subjected to high temperature treatment as a result of the different thermal properties of minerals, and even lead to changes in rock composition (such as high temperature melting and metamorphism), thus changing the physical and mechanical properties. In general, granite has a high ratio of compressive to tensile strength, and the tensile strength should receive adequate attention when designing high-level waste repository. The Ӓspӧ Pillar Stability Experiment (APSE) of HRL shows that the yield degree of rock mass is very sensitive to the small change of tangential stress, and the vast majority of fractures are tensile mechanisms^7^. Tensile strength plays a significant role in analyzing the failure mechanism of granite treated at high temperatures and designing of high-level radioactive waste repository. As shown in Table 1, the Brazilian tensile strength of granites with different mineralogical compositions after thermal treatment is summarized. Most recent experimental work found that Brazilian tensile strengths of most granites generally decrease after the heating–cooling cycle. In contrast, some studies found that tensile strength may not monotonically decrease as temperature increases to about 200°C. The thermo-mechanical behavior of granites under the Brazilian tests may be related to mineralogy, grain size, grain shape, etc. Some laboratory tests^14–17^ use acoustic emissions (AE), computed tomography (CT), and scanning electron microscopy (SEM) technologies to observe the micromechanism of thermal interaction of minerals, demonstrating that intergranular micro-cracks are formed first, followed by intragranular and transcrystalline ones. However, most of these studies focus on the statistics and imaging of thermal-induced micro-cracks due to the lack of effective methods to characterize the micromechanical state. It is still difficult to intuitively display the stress state of minerals treated by high temperatures. In other words, the relationship between heterogeneity of mineral thermal properties and mechanical response remains poorly understood.

**Table 1:** Summary of Brazilian tensile strength of granites after thermal treatment.

Rock type	Temperature (°C)	Mineralogical composition (%)					Average grain diameter	Tensile strength	b-value	References
		Fsp	Q	Bt	Pla	Other				
Yanshan granite	25–800	51	16	16	–	17	0.2–3.0 mm	Decrease	Decrease-increase–decrease	Guo et al.^15^
Heishantuo Granite	20–400	66	33	–	–	1	0.69 mm	Increase–decrease	–	Zhao et al.^18^
							0.85 mm	Decrease		
							1.39 mm	Increase–decrease		
Crystalline rock	30–350	–	–	–	–	–	–	Decrease	–	Roy and Singh^19^
Indian granite	30–160	48	39.5	1.5	10	1	⁓ 2.6 mm	Decrease	–	Dwivedi et al.^11^
Remiremont granite	30–600	22–24	25–27	5	41–45	3	0.5–2.0 mm	Decrease	–	
Salisbury granite	30–600	34–45	14–20	5–10	30–35	2–5	1.0–2.0 mm	Decrease	–	
Westerly granite	30–1050	31.4	29.3	3.8	31.3	4.2	–	Decrease	–	
Qinling biotite granite	25–1000	8	17	18	37	16	–	Decrease		Liu and Xu^20^

*Fsp Feldspar, *Q* quartz, *Bt* Biotite, *Pla* plagioclase.

## Brief description of modelling method

### Thermo-mechanical model description

**Figure 1:**
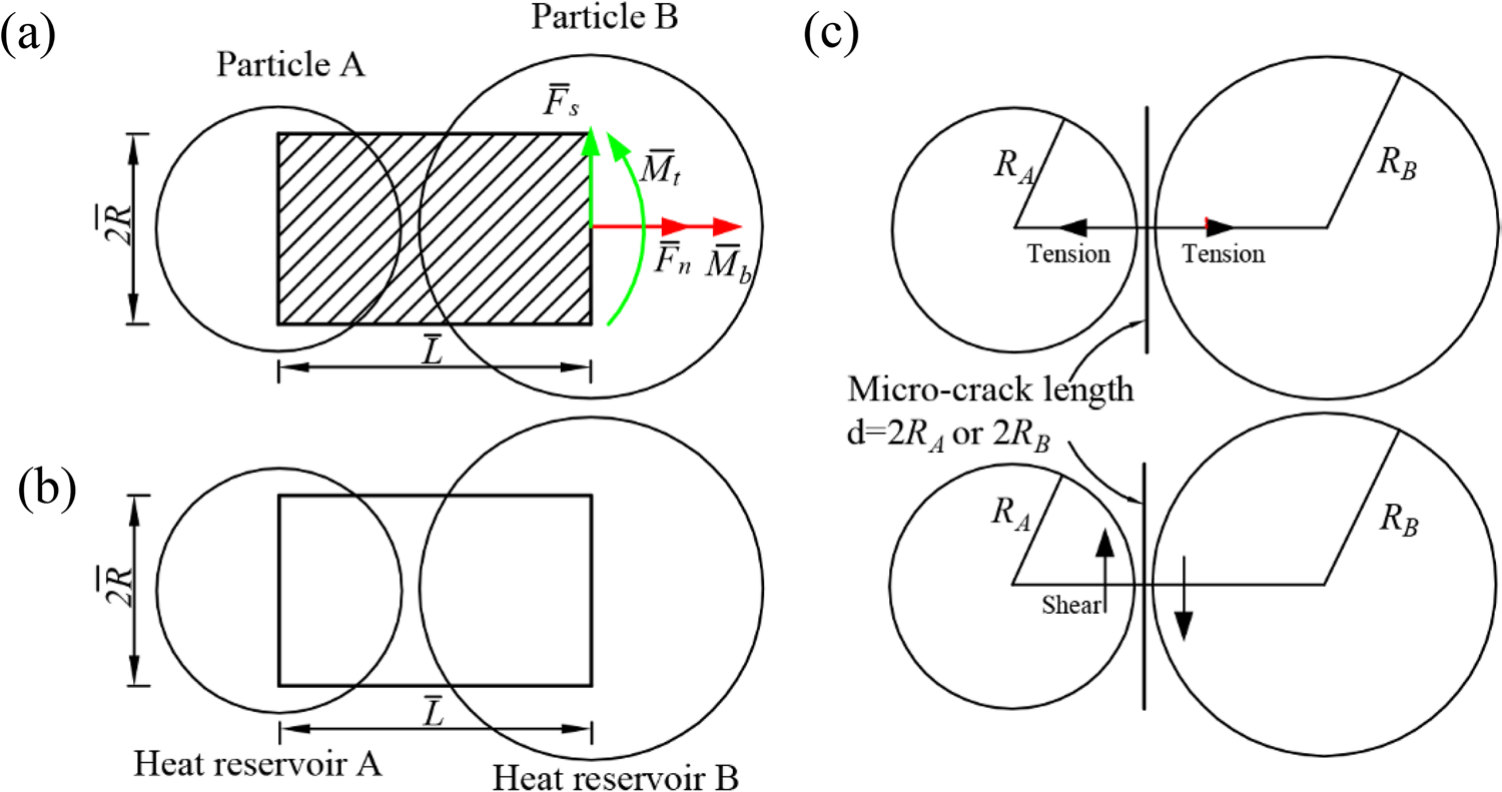
The thermo-mechanical model and the micro-crack definition: (**a**) the parallel bonded model; (**b**) thermal pipe contact model; (**c**) micro-crack definition after^21^.

Particle flow code (PFC) simulates the translation, rotation, and interaction of many particles, that may interact at bond contacts based on force and moment of internal faces. This method has been applied in many fields, including rock fracturing, excavation stability, and mining engineering^13,21–24^. The detailed effects of high temperature on the mechanical properties of Yanshan granite after the Brazilian test are simulated using the thermo-mechanical model. The mechanical model uses the parallel bond model and the thermal model uses the thermal pipe contact model, providing the behavior of an interface that carries force and moment (Fig. 1a) and a network of heat reservoirs and thermal pipes (Fig. 1b), respectively. The thermal pipe contact model contains three modified parameters, namely linear thermal expansion coefficient, thermal resistance, and temperature. By changing the grain size and the force carried in the bond, thermally induced strain is generated to simulate the heating of the grain and bonded material. This force and moment are bound up with maximum stresses acting within the bonded contact. The parallel bond breaks when the maximum normal or shear stress exceeds the bond strength (Fig. 1c). A detailed description of the parallel bond model and thermal pipe contact model follows Sun et al.^13,23,25^.

The maximum normal and shear stresses are defined as follows:

1 $$\overline{\sigma } = \frac{{\overline{F}_{n} }}{{\overline{A}}} + \beta \frac{{\left\| {\overline{M}_{b} } \right\|\overline{R}}}{{\overline{I}}}$$

2 $$\overline{\tau } = \frac{{\left\| {\overline{F}_{s} } \right\|}}{{\overline{A}}}{ + }\left\{ {\begin{array}{*{20}c} {0, 2{\text{D}}} \\ {\beta \frac{{\left| {\overline{M}_{t} } \right|\overline{R}}}{{\overline{J}}},3D} \\ \end{array} } \right.$$

where $\overline{F }$_n_ and $\overline{F }$_s_ denote the axial- and shear directed forces, respectively; $\overline{M }$_b_, $\overline{A }$, and $\overline{I }$ are bending moment, the area and the moment of inertia of the bond cross-section, respectively; $\overline{J }$ and *β* are polar moment of inertia of the parallel bond cross-section and the moment-contribution factor, respectively.

The definition of crack type is as follows: (1) Exceeding the tensile strength limit is defined as tensile crack ($\tilde{\sigma }$ ≥ $\tilde{\sigma }$_c_); (2) Exceeding the shear strength limit is defined as shear crack ($\overline{\tau }\ge \overline{\tau }$_c_), where the shear strength $\overline{\tau } = \overline{c} - \sigma \tan \overline{\phi }$.

By assuming that strain changes play a negligible role in influencing the temperature. The heat-conduction equation for a continuum:

3 $$- \frac{{\partial q_{i} }}{{\partial x_{i} }} + q_{v} = \rho C_{v} \frac{\partial T}{{\partial t}}$$

The relation between the heat-flux vector and the temperature gradient:

4 $$q_{i} = - k_{ij} \frac{\partial T}{{\partial x_{j} }}$$

Thermal strains of the parallel bond model account for the thermal expansion of the bonding material that joins them. The change each particle radius:

5 $$\Delta R = \alpha R\Delta T$$

We account for the expansion by assuming that the normal component of the force vector is carried by the bond. The normal component of the bond force vector:

6 $$\Delta \overline{F}_{n} = - \overline{k}_{n} A\left( {\overline{\alpha }\overline{L}\Delta T} \right)$$

where *ρ*, *Cv, q*_*i*_, *q*_*v*_ and *T* are the mass density, the specific heat at constant volume, the heat-flux vector, the volumetric heat-source intensity or power density, and the temperature, respectively; *k*_*ij*_ is the thermal-conductivity tensor; α is the thermal expansion coefficient; ${\overline{k} }_{n}$, *A and* $\overline{L }$ are the bond normal stiffness, area of the bond cross-section and bond length, respectively.

### Modelling acoustic emission and moment tensor inversion

Acoustic emission events (bond breakages) can be recorded when the microcracks occur in a specified space and time window in PFC. These microcracks are considered part of the same macro fracture AE event^26,27^. The moment tensor inversion is used to obtain the source mechanism of an event. This approach can resolve more complex mechanisms that might be associated with, for example, opening and closing cracks. A more detailed mechanism is displayed as the Hudson T-k plot allowing all events to be compared with each other and to know the source nature^28–30^. The isotropic **m**^**ISO**^ and deviatoric component **m**^**Deviatoric**^ describe the motion mechanism and volume changes at the source after moment tensor decomposition, which extract from the diagonalized moment tensor and composed the deviatoric eigenvalues *m*_i_*, respectively.

7 $$\begin{gathered} {\mathbf{m = m}}^{{{\mathbf{ISO}}}} {\mathbf{ + m}}^{{{\mathbf{Deviatoric}}}} {\mathbf{ = }}\left[ {\begin{array}{*{20}c} {M_{1} } & 0 & 0 \\ 0 & {M_{2} } & 0 \\ 0 & 0 & {M_{3} } \\ \end{array} } \right] \hfill \\ \hfill \\ \end{gathered}$$

*M*_ISO_, *M*_DC_ and *M*_CLVD_ are calculated as follows:

8 $$M_{Iso} = \frac{1}{3}\left( {M_{1} + M_{2} + M_{3} } \right)$$

9 $$M_{CLVD} = \frac{2}{3}\left( {M_{1} + M_{3} - 2M_{2} } \right)$$

10 $$M_{CLVD} = \frac{2}{3}\left( {M_{1} + M_{3} - 2M_{2} } \right)$$

11 $$R = \frac{{100 \times tr\left( {\text{M}} \right)}}{{\left( {\left| {tr\left( {\text{M}} \right)} \right| + \sum\limits_{i = 1}^{3} {\left| {m_{i}^{*} } \right|} } \right)}}$$

An event was defined as an implosion type(*R* ≤  − 30%), a tensile type(*R* ≥ 30%), and a shear type(− 30% ≤ *R* ≤ 30%)^31^. Ohtsu^29^ divided an event into a tensile, shear, or mixed one based on eigenvalues analysis. Because the implosive events are often recorded in situ as volume compensation occurs, the source mechanisms classified by^31^ are used in this study.

Furthermore, the values of Hudson *T-k* can be calculated from the moment tensor to visualize the complex micromechanisms^32^.

12 $$T = \frac{{2m_{2}^{*} }}{{\max (\left| {m_{1}^{*} } \right|,\left| {m_{3}^{*} } \right|)}}$$

13 $$k = \frac{{m^{ISO} }}{{\left| {m^{ISO} } \right| + \max (\left| {m_{1}^{*} } \right|,\left| {m_{3}^{*} } \right|)}}$$

**Figure 2:**
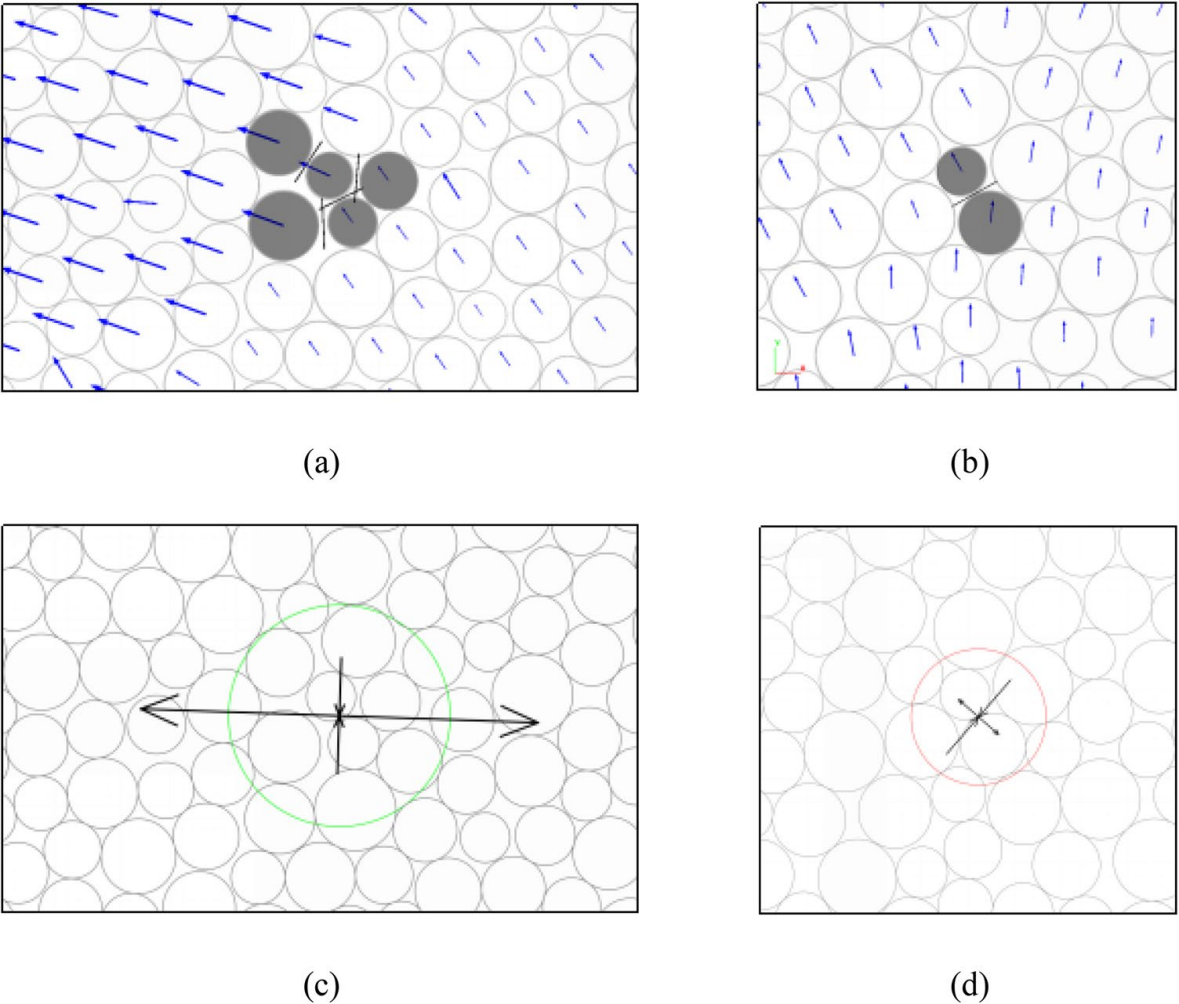
Examples of moment tensor representation method contains different micro-cracks. (**a**), (**b**) Source grains and micro-cracks (blue arrows, black line, and the darkened grain represent ball displacement, micro-crack, and source grain, respectively). (**c**), (**d**) The equal force and moment magnitude (green and red circles denote tensile and shear types).

Figure 2 shows an example tensile and shear source, which describes the situation 0.17 μs after parallel bonds break under uniaxial compression. The nature of the sources is identified as “tensile”, “shear” as suggested by Feignier and Young^31^. The moment tensor representation is described as two sets of arrows, where the length and direction of the arrows represent the equal force and orientation of the principal values of a moment tensor matrix, respectively.

## Modelling of Yanshan granite

### Experimental data details

**Figure 3:**
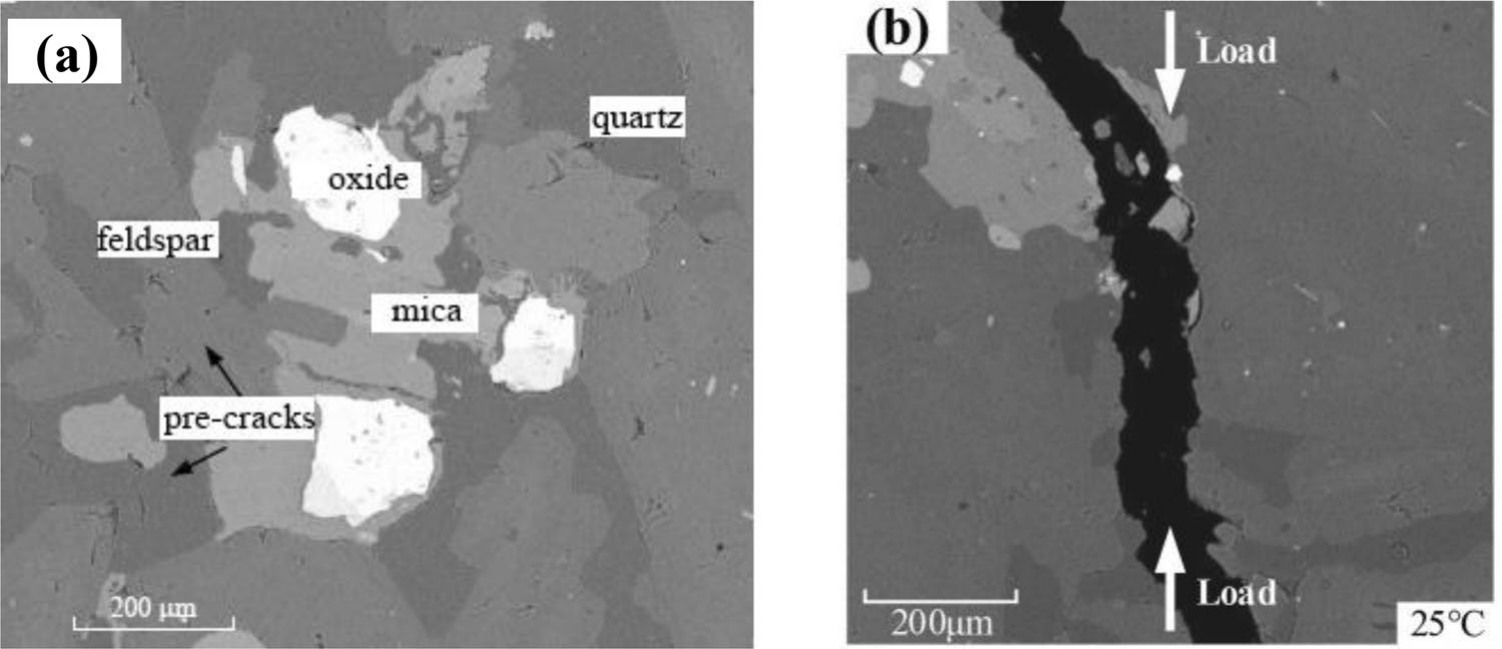
SEM images of Yanshan granite (**a**) at room temperature (25℃) and (**b**) after failure, modified after Guo et al.^15^, Wu et al.^33^.

To compare with laboratory tests, granite from Yanshan County, Jiangxi Province, China with uniform texture is selected in this study. Figure 3a shows the SEM image of Yanshan granite at room temperature. The mineral grain sizes range from 200 μm to 3 mm, comprising 32% biotite and hornblende, 30% albite, 21% orthoclase, 16% quartz, and other minerals. The Brazilian disc is 50 mm in diameter and 25 mm in thickness. The average density is 2800 kg/m^3^, and the porosity is 0.41%. The average uniaxial compressive strength and Brazilian tensile strength of granite are 196.56 and 10.594 MPa, respectively. The macro and micro-mechanical properties of Yanshan granite were achieved from a series of Brazilian tests with temperatures 25–800 carried out by Guo et al.^15^ and Wu et al.^33^. A typical SEM image of Yanshan granite after the Brazilian test is shown in Fig. 3b.

### The 2D parallel bonded model (PBM2D) specimen for the Brazilian test

**Figure 4:**
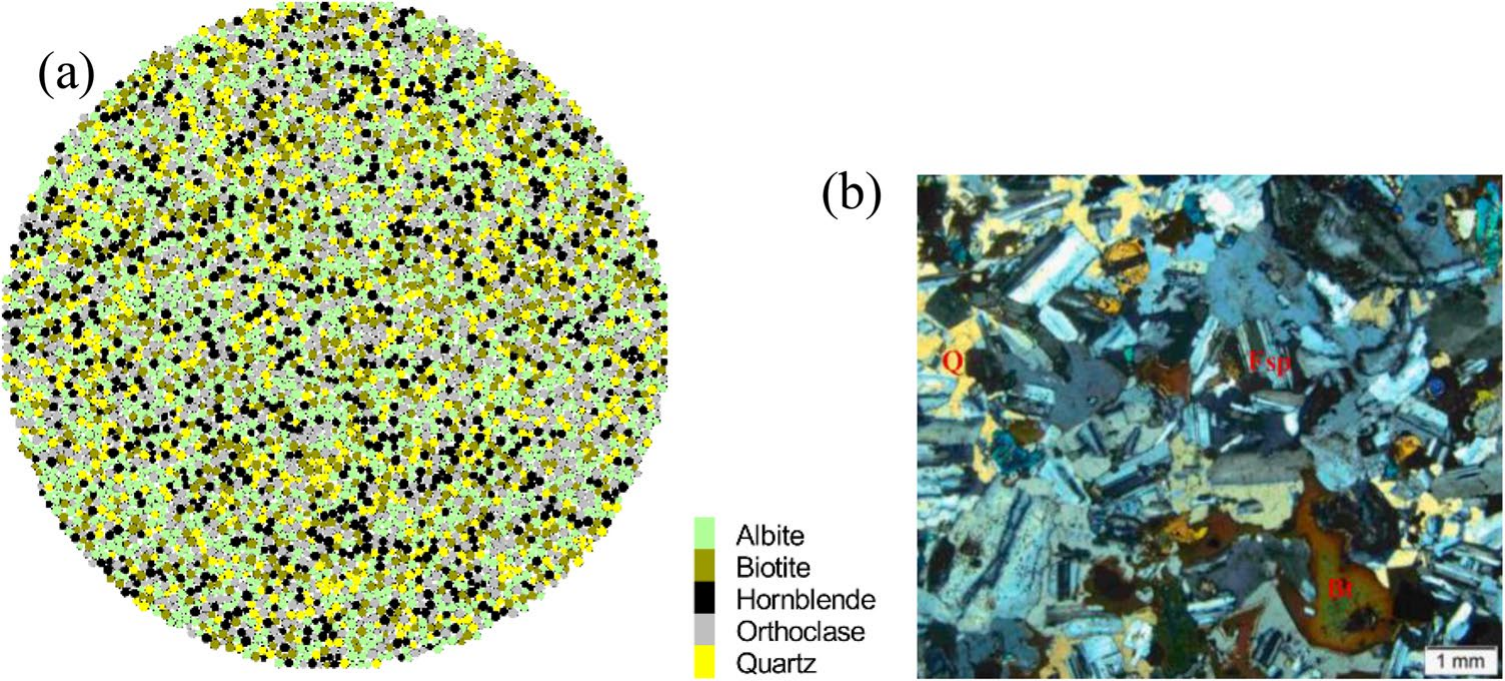
Yanshan granite specimen for thermo-mechanical coupling test: (**a**) thermally heterogeneous specimen, (**b**) experiment after Guo et al.^15^, Wu et al.^33^.

**Figure 5:**
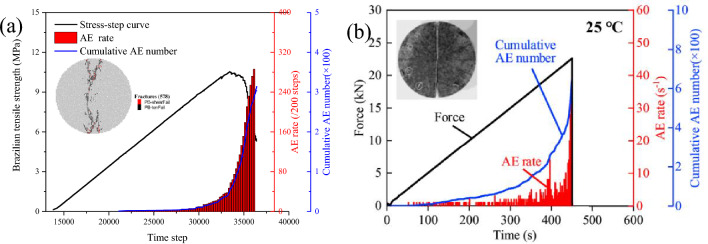
Stress, force, AE rate and cumulative AE number-step or time curves from (**a**) the simulation and (**b**) the laboratory test at room temperature after Guo et al.^15^, Wu et al.^33^.

The PBM2D sample follows a uniform distribution ranging from 0.5 to 0.83 mm, and the geometric dimensions of the sample have the same size as the experiments. The sample for the Brazilian test contains 5132 grains with 13,127 parallel-boned contacts. The grains are separated into five groups, ensuring that the particle ID number range of each group is consistent with the mineral proportion. Each group is randomly distributed with different thermal meso-properties, rather than particle clustered. The conceptual irregular shapes of particles are characterized by the real grain surface boundaries of granite, as shown in Fig. 4a. Figure 4b shows granite rock slices in the experiment for comparison. A trial-and-error method is used to calibrate the meso-parameters of numerical tests. The mechanical meso-parameters and the thermal meso-properties of the thermal pipe contact model are shown in Table 1. Using the meso-parameters in Table 2, the average Brazilian tensile strength of the numerical sample is 10.55 MPa, and the numerical sample well matches the Brazilian test behavior of experiments exhibiting a brittle load-time response, failure type, and AE characteristics (Fig. 5).

**Table 2:** Mechanical meso-parameters and thermal meso-properties for modelling Yanshan granite.

Model	Micro-parameters (unit)	Value
Parallel-boned model	Minimum grain radius (mm)	0.25
	The ratio of maximum to minimum grain radius (−)	1.66
	Installation gap (mm)	0.1
	Effective modulus (GPa)	18
	The ratio of normal-to-shear stiffness (−)	2.5
	Bond tensile strength, mean ± standard deviation (MPa)	23.2 ± 0
	Bond cohesion, mean ± standard deviation (MPa)	23.2 ± 0
	Friction coefficient (−)	0.5
	Friction angle (°)	0
Thermal pipe model	Thermal meso-properties^23^^,^^34^^,^^35^	
	Specific heat, *C*_*v*_ (J/kg K)	1015
	Thermal expansion coefficient, *a* (1/K)	Albite: 10.65 × 10^–6^ Orthoclase:9.7 × 10^–6^ Quartz: 24.3 × 10^–6^ Biotite: 3.0 × 10^–6^ Hornblende: 23.8 × 10^–6^
	Thermal conductivity, *k* (W/m K)	3.5

## Modelling results

### Mechanical behaviour of granite

#### Mechanical behavior of granite after heating/ heating- cooling cycle

**Figure 6:**
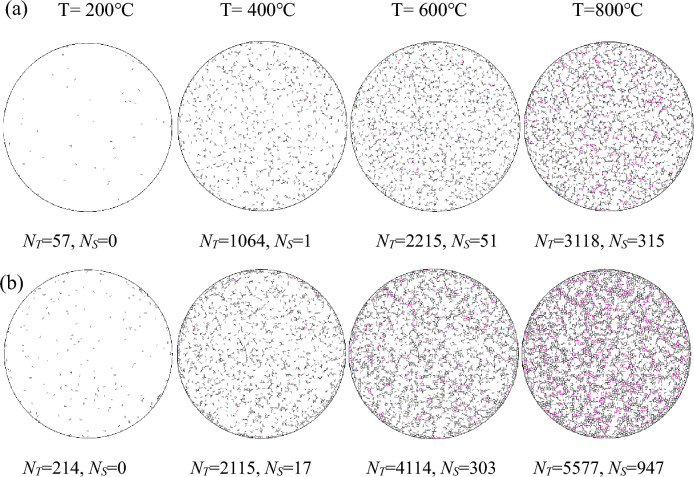
The distribution of thermally induced micro-cracks during (**a**) heating and (**b**) heating–cooling processes (the black and purple lines represent tensile and shear micro-cracks,* N*_*T*_ and* N*_*S*_ denote the number of tensile and shear micro-cracks).

Figure 6 shows the distribution of thermally induced micro-cracks recorded from the heating and heating–cooling processes. During the heating process, the distribution of thermally induced tensile micro-cracks is sparse at T = 200 °C. The clusters of thermally induced micro-cracks appear around the initial micro-cracks, and thermally induced tensile micro-cracks increase dramatically at T = 400 °C. With the temperature increasing to 600 °C and 800 °C, the tensile micro-cracks further increase rapidly and shear micro-cracks are generated due to some particles slipping. These microcracks are very close, and some can be regarded as the generation of macro-cracks. The thermal-induced damage has no main direction, and the accumulated damage can not be recovered. At first, the thermal micro-cracks occur because the local stress concentration caused by the heterogeneity of thermal properties exceeds the contact bonded strength. Then, the stress concentration phenomenon is generated at the tip of the micro-crack according to Griffith’s theory^36^. The thermal stress generated by further heating causes the tip of the microcrack to further expand, resulting in a sharp increase in micro-cracks owing to the interactive effect between thermally induced micro-cracks and stress. During the heating–cooling process, additional tensile and shear micro-cracks can be generated during cooling. The number of thermal micro-cracks increases rapidly and shows a positive relationship with temperature during the cooling process of 400–800–25 °C. Due to the combined action of micro-cracks and thermal stress caused by particle shrinkage during cooling, the particle stress exceeds the bond tensile or shear strength causing new micro-cracks to be generated.

**Figure 7:**
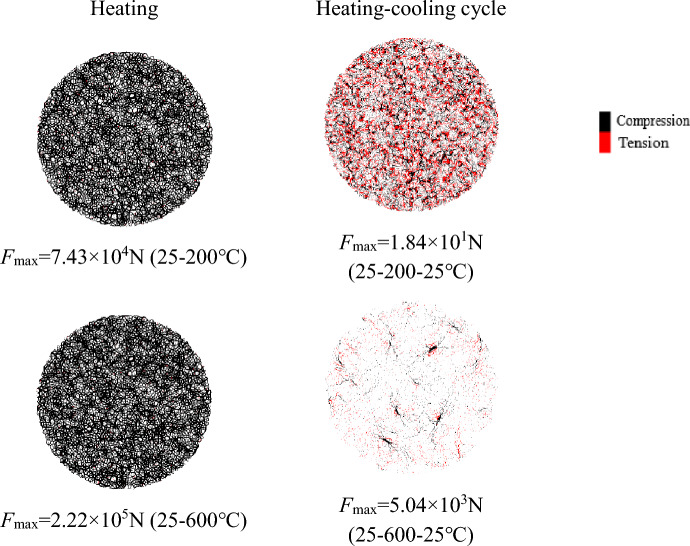
Distribution of thermal stresses after heating and heating–cooling processes (the black and red cylinders denote compressive and tensile force, respectively).

The distribution of the thermal stresses (contact force) after heating and heating–cooling processes under different temperatures are shown in Fig. 7. The cooling process will not restore the bonding force to the state before heating, and will produce residual thermal stress. The maximum force increases from 7.43 × 10^4^ N to 2.22 × 10^5^ N during heating of 25–200–600 °C during the heating process, indicating that the maximum contact force increases with temperature during the heating process. The cooling process greatly affects the distribution and degree of stress concentration. During the cooling process, the residual maximum force of the numerical granite sample increases from 1.84 × 10^1^ to 5.04 × 10^3^ N during cooling of 200–600–25 °C, demonstrating that the residual maximum force increases with increasing temperature. The distribution of the maximum bonding force after higher temperature treatment presents sparse local stress concentration during the cooling process. The contact type is mainly compression during the heating process, while mainly tension and compression during the cooling process. The cooling process does not return to the original state according to the force and deformation path of the heated particles due to the interaction effect of the thermal cracking and the free expansion of the boundary.

**Figure 8:**
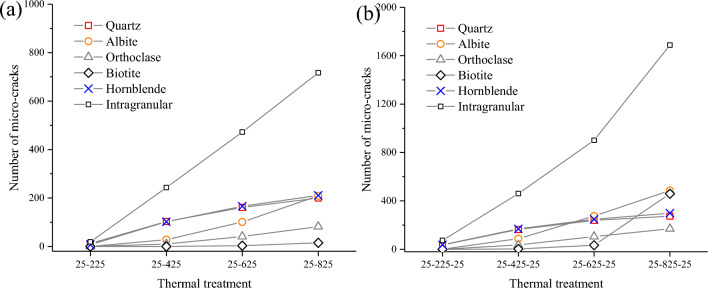
The number of intergranular and intragranular micro-cracks after (**a**) heating and (**b**) heating–cooling processes.

**Figure 9:**
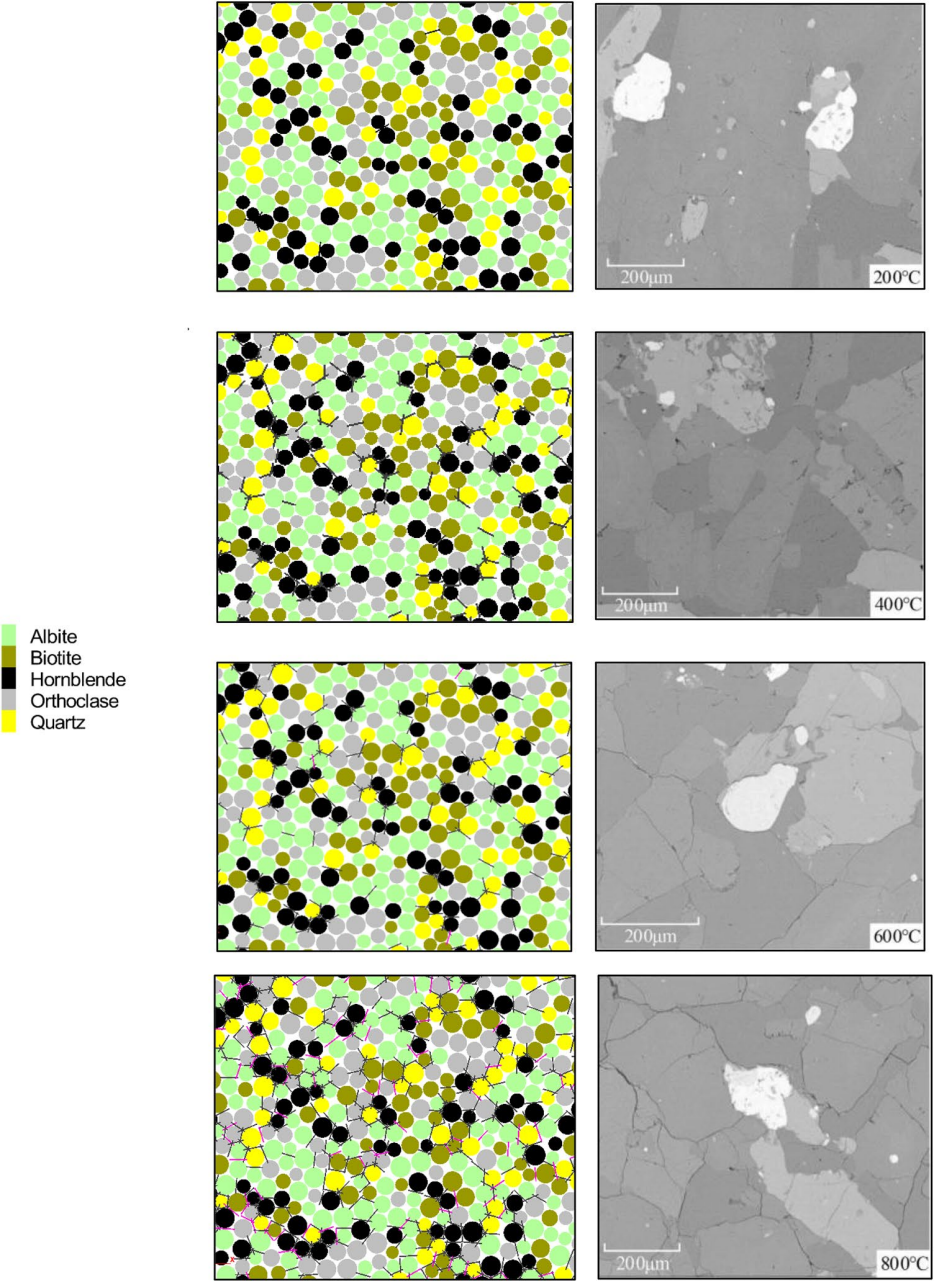
The distribution of intragranular, intergranular and transcrystalline micro-cracks from simulations (*left*, T = 200, 400, 600, 800 °C) and laboratory tests after heating–cooling process. (*right*, T = 200, 400, 600, 800 °C, modified after^15^,^33^.

The number of intergranular and intragranular micro-cracks during heating and heating–cooling processes are accounted for Fig. 8. The results show that the intergranular ones generated at the interfaces between minerals with different thermal expansion coefficients play a dominant role. During the heating process, the intragranular ones predominate in quartz and hornblende with a greater coefficient of thermal expansion, followed by albite, orthoclase, and biotite with smaller coefficients of thermal expansion. During the heating–cooling process, the intragranular micro-cracks predominate in albite and biotite during the heating of 25–600 °C and 25–800 °C due to their high mineral content. Figure 9 shows the distribution of intragranular, intergranular, and transcrystalline micro-cracks in the simulation, and qualitatively compares it with the SEM photos of granite specimens. For numerical results, there are mostly intergranular and intragranular micro-cracks at low temperatures (T = 200, 400 °C), while numerous transcrystalline ones appear at higher temperatures (T = 600, 800 °C). This sequence is quite analogous to the laboratory test, that is, intergranular and intragranular micro-cracks are generated first, and then transcrystalline ones are generated.

#### Mechanical behaviour of granite after thermal treatment

**Figure 10:**
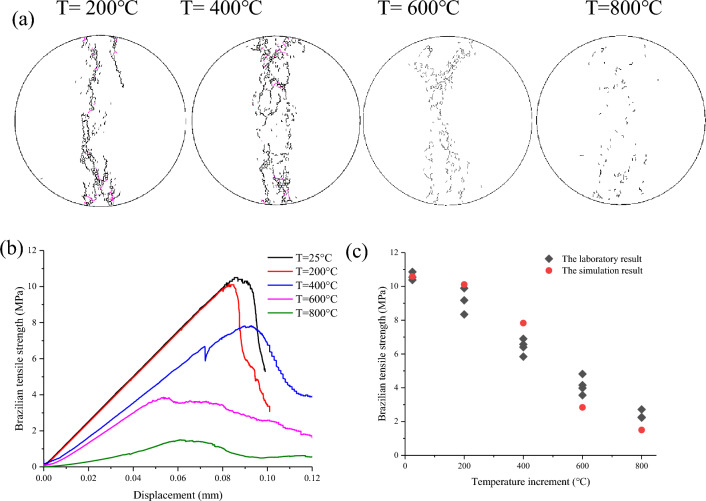
The results of (**a**) the damage pattern, (**b**) the Brazilian tensile strength-displacement curve, and (**c**) the comparison with laboratory results after different heating–cooling cycle.

The temperature effects on the damage pattern and Brazilian tensile strength of granite are presented in Fig. 10. The results indicate that the macroscopic failure model could be changed with increasing temperature. The macroscopic single fracture occurs parallel to the load direction, forming the failure band relatively narrow at low temperature (T = 200 °C), which is a typical failure model of transition between shear-tensile failure at the ends of the specimen and tensile failure mainly along the diameter. The macroscopic multi-fractures occurred along the loading direction, resulting in a wide failure band at high temperatures (T = 400, 600, 800 °C). The tensile strength exhibits a monotonous decreasing trend with increasing temperature. The Brazilian tensile strength is 10.59 MPa at room temperature (25 °C), while Brazilian tensile strengths treated at 200 °C, 400 °C, 600 °C, and 800 °C decrease to 10.11, 7.83, 2.84, and 1.50 MPa, respectively. The average Brazilian tensile strengths of laboratory tests change to 9.18, 6.43, 4.13 and 2.40 MPa at 200 °C, 400 °C, 600 °C, and 800 °C, respectively. Both the numerical simulations and laboratory tests exhibit an obvious reduction in Brazilian tensile strength after high thermal treatment (≥ 400 °C), as shown in Fig. 10c. This trend is similar to Shao et al.^37^, who found the compressive strength of Strathbogie granite decreased with temperature up to 400 °C. This trend is also similar to Zhao et al.^18^, and Roy and Singh^19^, who found the tensile strength of crystalline and Heishantuo granite has a significant reduction when the holding temperature was up to 400 °C. With the increase in temperature, the rock specimen gradually transits from brittle splitting failure to ductile failure, and the strain before failure also gradually increases in many uniaxial and triaxial tests^38^,^16^. The Brazilian tensile strength-displacement curve indicates that the Brazilian disc after high temperature treatment also conforms to this rule. In general, the higher temperature generally encourages ductility and decreases tensile strength as a result of the interaction effect of thermal stress and thermally induced micro-cracks.

**Figure 11:**
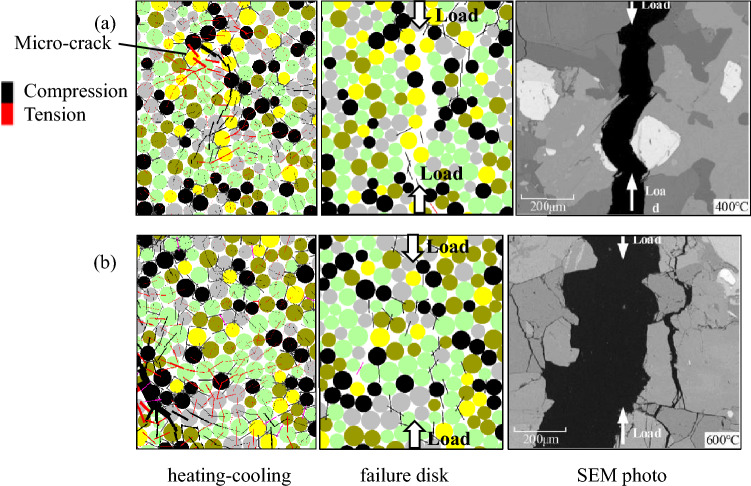
The local micro-cracking perspective of Brazilian disks subjected to (**a**) 400 °C and (**b**) 600 °C (SEM photo after Guo et al.^15^).

Figure 11 shows the comparison of a local micro-cracking perspective of Brazilian disks subjected to 400 °C and 600 °C between the numerical simulations and laboratory tests. On the left is the local contact force and thermally induced micro-cracks during the heating–cooling process. The middle and right figures are failure disks subjected to Brazilian tests after thermal treatment, which contain part of the macro-fractures of the disks. There are many micro-cracks near the main macro-fracture of the discs treated at high temperatures, showing a high degree of fragmentation. This is similar to the results observed by SEM. The increase in the density of thermal micro-cracks mainly contributes to cracking behavior during the Brazilian test, and thermal stress after heating–cooling cycle also plays a role.

**Figure 12:**
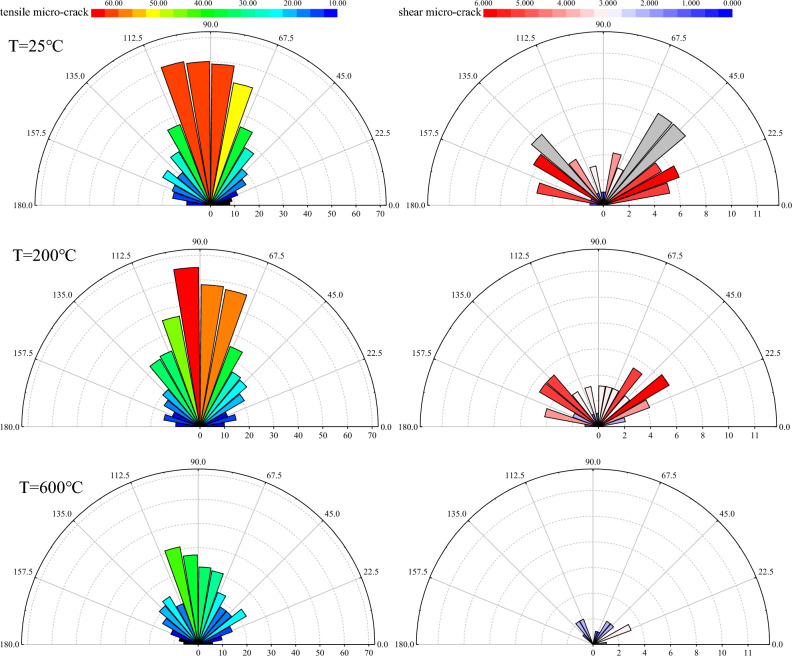
Orientation distribution of tensile (left column with share color bar of tensile micro-crack) and shear (right column with share color bar of shear micro-crack) micro-cracks after thermal treatment.

Figure 12 shows the effect of thermal treatment on the orientation distribution of micro-cracks in Brazilian tests. The distribution of tensile and shear micro-cracks was statistically analyzed using a rose plot, with the circumferential number representing the orientation and the radial one representing the frequency of micro-crack occurrence. The orientation of tensile micro-cracks is mainly parallel to the axial loading direction, and shear micro-cracks are mostly inclined around 45℃ or 135 °C. The frequency of the two types of micro-cracks significantly decreases with increasing temperature, while the dominant orientation remains significant. As the temperature increases, the concentration of tensile micro-crack orientations near 90 °C decreases. This is mainly due to the formation of rock bridge structures by thermally induced micro-cracks, changing orientations of some tensile micro-cracks that may have previously occurred parallel to the loading direction. The orientation of shear micro-cracks changes with temperature, mainly manifested by a decrease in the dominant orientation angle, indicating that more particle sliding with a low angle occurred in the Brazilian disc after high temperature. This may be related to the increased ductility of the sample after high temperature.

### AE characteristics

#### AE characteristics of failure process

**Figure 13:**
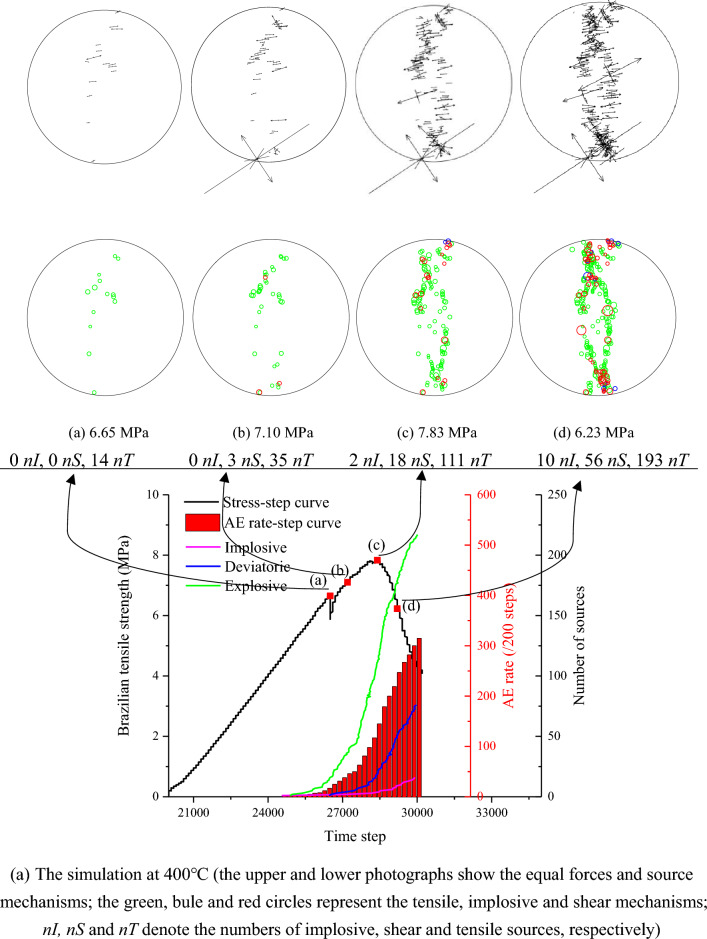

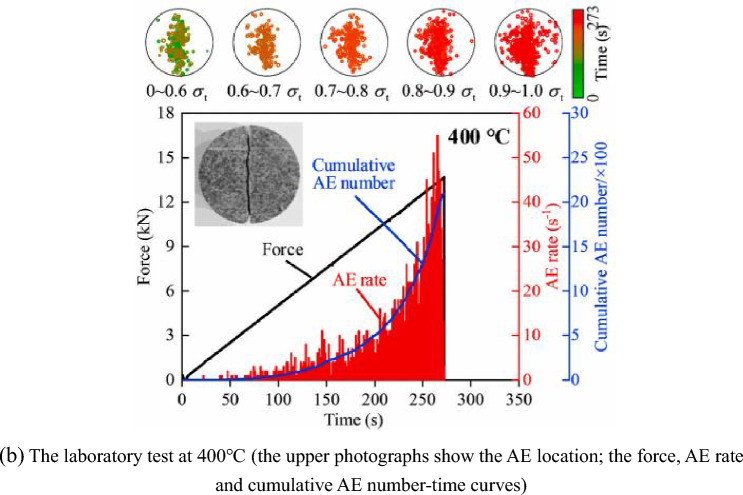
The failure process of (**a**) the simulation at 400 °C, and (**b**) laboratory test at 400 °C after Guo et al.^15^.

This failure process can be further represented by calculating the moment tensor. Figure 13a gives the development of the equal forces and source mechanisms of the Brazilian disks subjected to 400 °C. The visualization of the equal forces, and source mechanisms is plotted for all events. The moment tensor and moment magnitude are displayed as equivalent forces and circle radius, respectively. The two sets of arrows pointing away represent a tensile source, while two equal-length arrows pointing in opposite directions represent a shear source. The failure process can be divided into the following four stages:

- Crack initiation: at this time, the stress is approximately 85% of the peak stress. The crack initiation is close to the center of the disc, and the AE events are scattered. The sources are all tensile, implying that the tensile forces of the tensile effect play a dominant role.
- Crack propagation: the events further accumulate near the center of the disc, indicating that some macro-cracks begin to appear. The nature of the source is still predominated by tensile opening vertical load direction. In addition, some shear sources occur along the loaded diameter, which is oriented at about 45° to the vectors.
- Crack coalescence: the load is approximately 100% of the peak stress and micro-cracks coalesce along the loading direction. The possible failure zone is parallel to the loading direction and the direction of the tensile events near the center of the disk is almost completely horizontal. The failure nature is still a high proportion of tensile sources, but shear source also plays a role.
- Post-peak failure: the number of three natures of sources increases rapidly in a short time step after peak stress, causing catastrophic crushing. After the peak stress, the number of three sources rapidly increases in a short period, resulting in catastrophic crushing. When the compression component exceeds the explosion component, a small number of sources showing the implosion mechanism appear at both ends of the sample. The nature of the source is predominated by tensile and shear. There is a phenomenon of scattered events distribution of disk subjected to higher temperature.

The numerical simulation results are consistent with the laboratory test results. Figure 13b shows the failure process of the laboratory test at 400 °C, indicating that the AE rate and cumulative AE number show an increasing trend as the load increases. After high temperature, the distribution of the events is mainly concentrated in the axial direction near the center, and there are also a certain amount of micro-cracks on both sides of the deviation from the central axis. This is mainly due to the local stress concentration caused by thermal damage, which leads to fractures under small external forces in the noncentral region.

#### The micromechanics of the source

**Figure 14:**
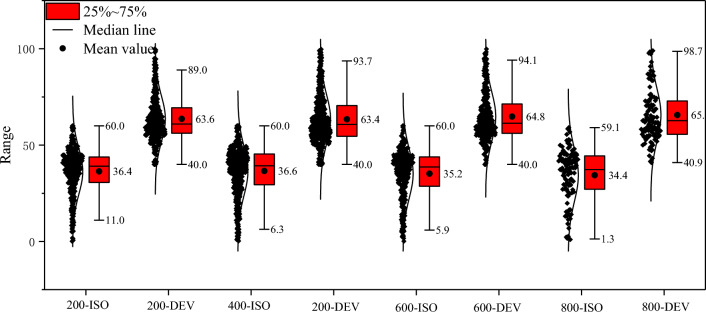
The variation of proportion of ISO and DEV components with temperatures.

To further quantify the changes in the mechanism of seismic sources, statistical analysis was conducted on the isotropic (ISO) and deviatoric (DEV) components. The statistical results are presented in a box chart, which includes the maximum value, minimum value, average value, and distribution of data points, as shown in Fig. 14. The isotropic (ISO) component that denotes the explosion or implosion volume change. The deviatoric component (DEV) contains double couple (DC) and compensated linear vector dipole (CLVD) components that denote the amount of shear dislocation and the compression with no volume change, respectively. The maximum value of ISO composition remains within the range of 200–600 °C at approximately 60% and decreases from 800 °C degrees to 59.1%. The minimum ISO value ranges from 1.3 to 11.0%, decreasing with increasing temperature. The average ISO value ranges from 36.6 to 34.4%, showing a trend of decreasing after increasing in a small range. The maximum value range of DEV composition is between 89 and 98.7%, which increases with temperature. The minimum value of DEV composition remains within the range of 200–600 °C at approximately 40% and increases from 800℃ degrees to 40.9%. The average DEV value ranges from 63.4 to 65.1%, showing a trend of increasing after decreasing in a small range. ISO is represented by three equal eigenvalues matrices, namely the center of expansion or compression, and positive values can to some extent reflect the tensile micromechanism. This result indicates that the tensile mechanism decreases with the increase of temperature. DEV represents a partial component, which to some extent represents shear failure or the relative dislocation micromechanism of faults. The results show that the shear micromechanism increases with the increase in temperature.

**Figure 15:**
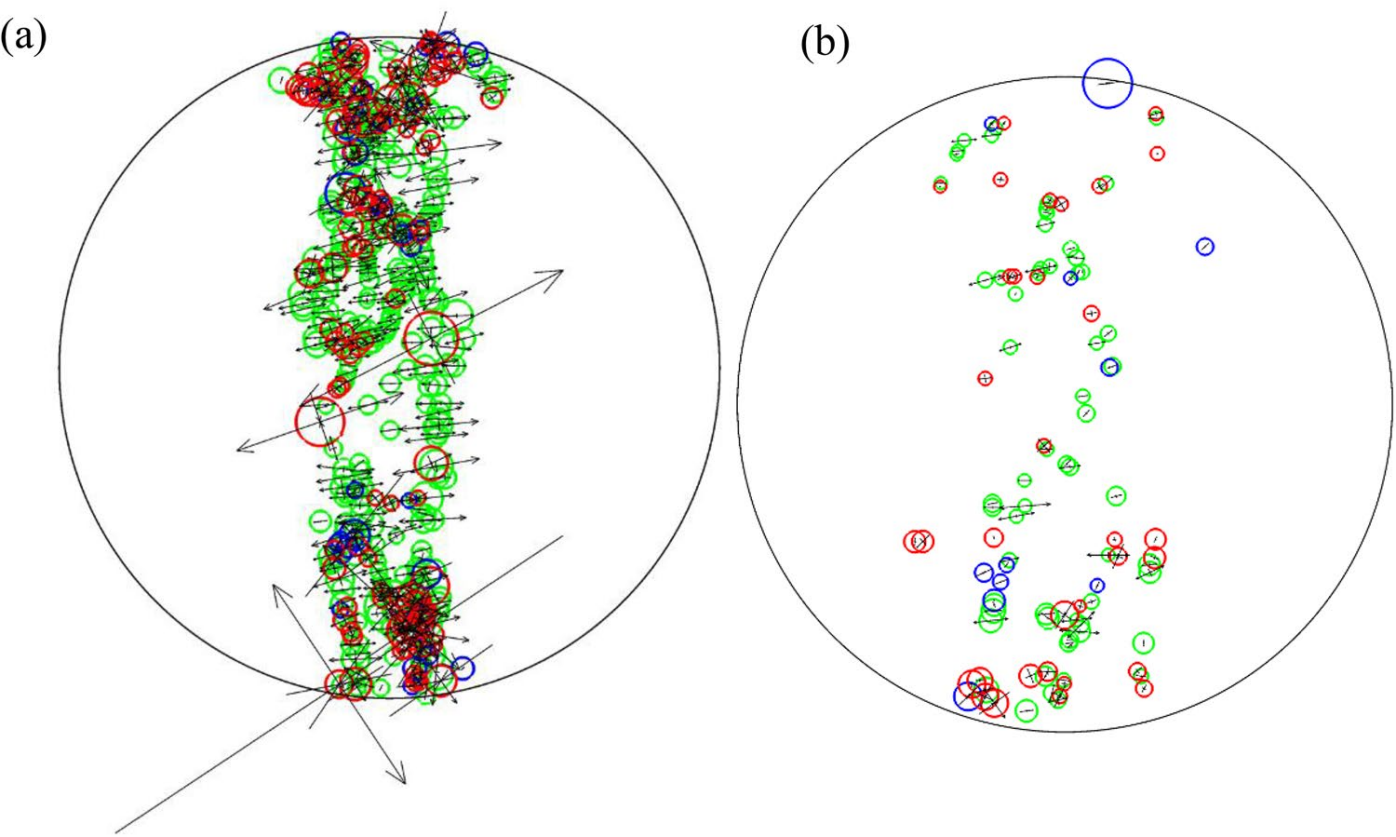
Comparison of source mechanisms between (**a**) T = 400 °C and (**b**) T = 800 °C.

Further comparison of the source mechanisms and moment tensors of Brazilian tests at different temperatures. The parameters to be compared include magnitude, the nature of the source, size, and direction of equivalent force, and magnitude distribution, as shown as in Fig. 15. High temperatures significantly reduce the magnitude, number, and equivalent force of seismic sources. The source distribution is more dispersed at 800 °C than 400 °C, and more shear mechanics occur on the nonloaded axis.

**Figure 16:**
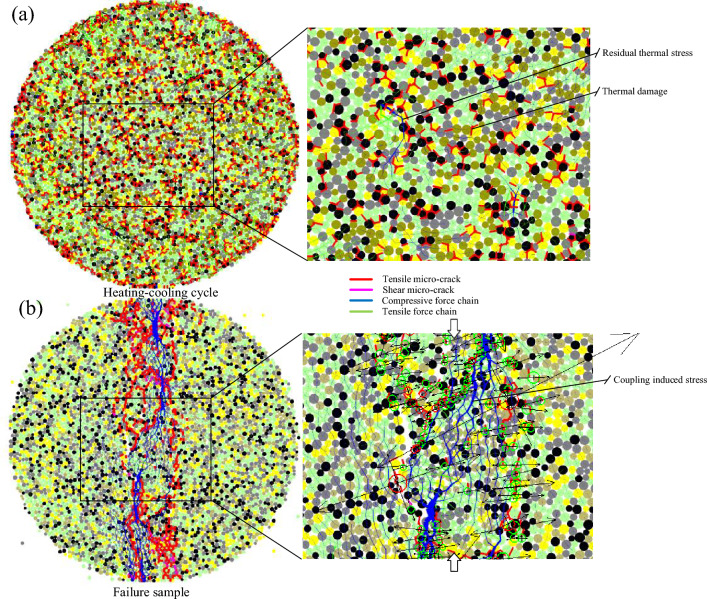
The micromechanics of the source by coupling induced stress at 400 °C: (**a**) after heating–cooling cycle, and (**b**) after Brazilian test.

More detailed microscopic mechanisms can be further presented through local observations. Figure 16 takes the T = 400 °C as an example, containing data such as grain composition, microcracks, force chains, magnitude, and other forces. After the heating–cooling cycle, intergranular, intergranular, and transgranular microcracks are generated inside the sample, causing thermal damage. At the same time, residual thermal stress still exists inside the sample after cooling. Its distribution is the result of the interaction between thermal paths, grain distribution, and microcracks. As the axial stress applied by the loading plate is transmitted to the particles, the area where thermally induced microcracks and residual thermal stress are concentrated in the axial direction is most likely to initiate cracking. In areas with less thermal damage, fracture mainly occurs due to open. Particles near the concentration of thermal damage are prone to sliding along thermal-induced microcracks under stress, resulting in shear micromechanics.

**Figure 17:**
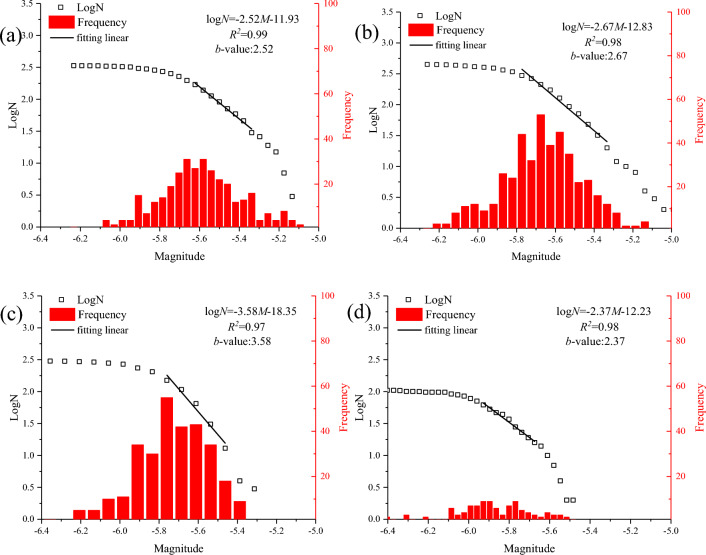
The relationships between *b* value and temperatures. (**a**)–(**d**) T = 200–800 °C.

The energy release is evaluated by the Gutenburg-Richer relationship between the logarithm of the event cumulative number (LogN), frequency, and magnitude^39^, as shown in Fig. 17. The b-value is generally used to measure the relative numbers of small and large events, which is thought to be as an indicator relating to variations of the stress state. The *b*-value ranges from 2.37 to 3.58 by fitting the straight part of the data, showing a positive and then negative relationship with the increase of the temperature. In general, the slope of the curve is steeper, indicating that fewer large-magnitude sources during the Brazilian test. There are fewer thermal-induced micro-cracks at low temperature (T = 200 °C). The more parallel bond breakages occurring within a specified time and space window are part of one event during loading, resulting in more large magnitude events (low *b*-value). There are more thermally induced micro-cracks as temperature increases (T = 400 and 626 °C). The fewer parallel bond breakages occurring within a specified time and space window form small magnitude events, contributing to the high *b*-value. There is also a level-off part in the distribution of magnitude and frequency as temperature reaches 800 °C. The number of thermal micro-cracks increases extremely at 800 °C, and the external force required for sample destruction drops to a very low level. Thermal damage reduces the macro-cracking process due to the mechanical degradation of the sample, which results in the occurrence of more small-magnitude making the *b*-value relatively lower. The experimental results of target granite on the *b*-value treated at 200 °C, 400 °C, 600 °C, and 800 °C are 1.283, 1.193, 1.389, and 1.360, respectively, displaying a increasing trend as the temperature increases^15^. Akdag et al.^40^ found that the trend of *b*-value increases first and then decreases with increasing temperature, which is consistent with the numerical simulations. The value of target granite is larger in experiments, which is a limitation of this study. The reason may be due to the loss of small magnitude events caused by a setting trigger threshold for the gain channels to trigger the continuous waveforms.

### Statistics results of source mechanism

The different classification methods of source mechanism based on moment tensor inversion are used to classify the events in the Brazilian tests after thermal treatment, and the statistical results are shown in Table 3. The classification method proposed by Feignier and Young^31^ shows that the proportion of tension is more than 50%, followed by shear and implosive sources. The proportion of tensile sources decreases from 65.1 to 57%, the proportion of shear sources increases from 25.7 to 31.8%, and implosive sources with a small increase in proportion (about 10%) with increasing temperature from 200 to 800 °C. Thermal damage affects the nature of sources due to the mechanical degradation of the samples, showing a decreasing of tensile source and an increasing shear source. The method proposed by Hudson et al.^32^ shows that the average value of T ranges from − 0.55 to − 0.38, and k ranges from + 0.17 to + 0.21, which is located in the upper left “quadrant” of the T-k plot, showing that tensile sources are not pure tensile fractures, but also contain some deviatoric component. Therefore, the fracture mechanism of the Brazilian test with high temperature treatment can be explained as mainly tensile, accompanied by a small amount of shear fracture. The experimental results of target granite have no data related to the source mechanism, which is a limitation of this study.

**Table 3:** Statistical results of source mechanism using different classification methods.

Thermal treatment ( °C)	Source types by Feignier and Young (1992)			Hudson T-k by Hudson et al.^32^	
	Implosive	Tensile	Shear	Mean T	Mean *k*
T = 25	29 (9.2%)	205 (65.1%)	81 (25.7%)	− 0.46	+ 0.20
T = 200	25 (7.5%)	233 (69.1%)	79 (23.4%)	− 0.49	+ 0.21
T = 400	36 (8.0%)	296 (65.9%)	117 (26.1%)	− 0.55	+ 0.20
T = 600	34 (11.2%)	189 (62.2%)	81 (26.6%)	− 0.43	+ 0.18
T = 800	12 (11.2%)	61 (57%)	34 (31.8%)	− 0.38	+ 0.17

## Discussion

**Figure 18:**
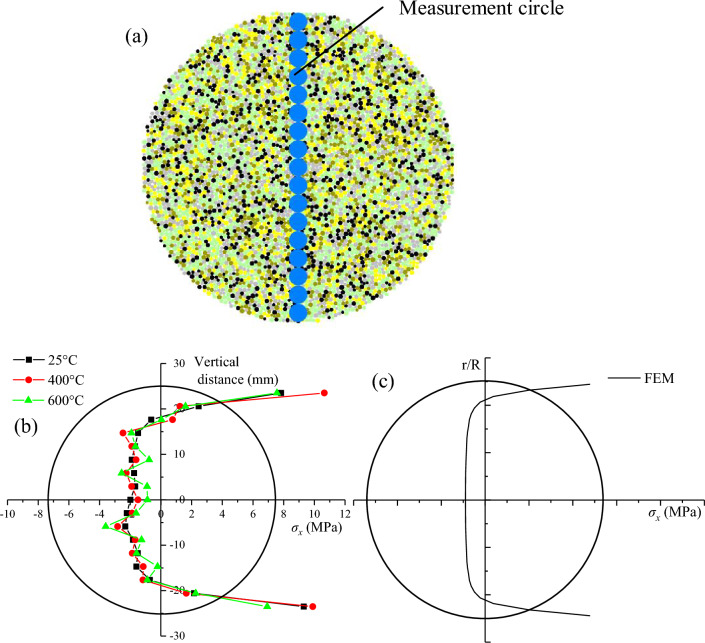
Stress state analysis of Brazilian disk: (**a**) the arrangement of measurement circles; (**b**) the distribution of horizontal stress treated at 25 °C, 400 °C, 600 °C; (**c**) the continuum method after Li and Wong^41^.

To further investigate the effects of the thermally induced micro-cracks and thermal stress on the initiation point of the Brazilian disk, 17 measurement circles are symmetrically installed along the compressive diametral line to record the horizontal stress (Fig. 18a). It’s worth noting that the measured horizontal stress is the average stress, as each measurement circle contains 4–6 particles. Figure 18b shows the distribution of horizontal stresses when the tensile strength reaches 2.0 MPa with few micro-cracks induced by loading. As shown in Fig. 18c, the distribution of horizontal stress recorded in PBM simulation has parallels in the continuum method. The horizontal stresses within a certain range extending from the center of the disk to the load points are in a tensile state, while the horizontal stresses near the load points transition to a compressive state. Nevertheless, the horizontal stress changes rapidly along the compressive diametral line, casting the micromechanisms of the Brazilian disc after high temperature in a new light. Under the same load condition, the fluctuation degree of horizontal stress increases with the increase of temperature. At low temperature, there are few thermal micro-cracks, and the fluctuation of the horizontal stress of the measurement circle is mainly affected by the thermal stress component. For the high temperature treated sample, the measuring circles contain some thermally induced micro-cracks and thermal stress, resulting in a greater degree of fluctuation of the horizontal stress. From the change of fluctuation degree, it can be concluded that thermal damage plays a dominant role in the influence of horizontal stress. The degree of sample heterogeneity is increased, and some singular points appear far away from the theoretical analysis as the influence of thermally induced micro-cracks and thermal stress. Thus, it is concluded that the non-central initiation of the Brazilian disk can be caused by thermal damage and thermal stress.

The multi-mineral composition sample established by this research institute ensures consistency with laboratory tests in terms of mineral composition proportion and thermal parameters and forms complex grain boundaries in the form of random distribution. However, the irregular shape of the grains in the numerical model does not match the mineral grain boundaries of natural marble. The numerical model uses contact strength micro-parameters with standard deviation to simulate the different bonded strengths between natural mineral grains, but it still cannot accurately match the bonded state of natural marble. The meso-parameters of the contact model between different mineral grains are not strictly set according to the actual different types of contact grains (intragranular and intergranular contacts), and this study only calibrates the macroscopic parameters. One possible improvement method is to use Voronoi partitioning to divide the construct grains into groups. The calibration of grain contact and thermal parameters by obtaining the shape of grain boundaries, using different mechanical contact models (intergranular and intragranular contact), and assigning corresponding thermal parameters to different particles using a heat pipe model. In the future, it can be considered to use this method to simulate geometric boundaries based on natural rock grain boundaries and assign different grain-to-grain bonded micro-parameters.

## Conclusions

To understand the roles of high temperature in weakening granite and thermally induced micro-cracking process in the Brazilian test, the thermo-mechanical model based on bonded particle model and moment tensor inversion is used to quantitatively evaluate the thermo-mechanical characteristics of granite with mineralogical compositions. The simulation results match well with those measured from laboratory tests, and the failure process of the Brazilian disc treated by high temperature is analyzed in detail at the microscale. The main conclusions are as follows:

1. During heating and heating–cooling processes, the intergranular tensile micro-cracks at the interfaces between different minerals with different thermal expansion coefficients play a dominant role, followed by intragranular tensile ones mainly distributed in minerals with greater thermal expansion coefficients.
2. The Brazilian tensile strength and failure model of granite could be changed with increasing temperature. The Brazilian tensile strength exists a critical temperature, exhibiting an obvious reduction when the treated temperature is up to 200 °C. The results of orientation distribution, cracking behavior, and horizontal stress distribution indicate that the crack initiation and failure model is related to the heterogeneity caused by the thermal damage and thermal stress.
3. The damage evolution and nature of the source are quantitatively identified based on the* b*-value, the components of the moment tensor, and T-k values during the loading process. The *b*-value (energy change) increases and then decreases with increasing temperature. The fracture mechanism of the Brazilian tests with high temperature treatment can be explained as mainly tensile source (positive volume change), accompanied by a small amount of shear source (negative volume change). With the increasing temperature, the proportion of shear source increases due to the effect of initiation, propagation, and coalescence of thermally induced micro-cracks.

This study provides some new insights into the mechanical behavior of granite treated at high temperature. Granite, as the surrounding rock for the high-level nuclear waste repository, not only needs high strength but also suggests that its mineral composition should have relatively uniform coefficients of thermal expansion. The energy release and event frequency are affected by temperature, and the precursory information of granite failure is significantly different from that at room temperature. The tensile strength measured by the Brazilian test after high temperature has the problem of non-central initiation and increasing proportion of shear cracks, which may lead to higher measured results.

## Data Availability

The datasets generated and/or analysed during the current study are available in the figshare repository, 10.6084/m9.figshare.25325335.
